# Experimental study of radiation shielding performance of PbO_2_-BaO-CaO-B_2_O_3_-Y_2_O_3_ glass systems

**DOI:** 10.1038/s41598-026-39038-w

**Published:** 2026-03-09

**Authors:** M. Elsafi, M. I. Sayyed, Shams A. M. Issa

**Affiliations:** 1 Physics Department, Faculty of Science, Alexandria University, Alexandria, Egypt; 2 Department of Physics, Faculty of Science, Isra University, Amman, Jordan; 3 Department of Physics, Dogus University, 34775 Istanbul, Türkiye; 4 Physics Department, Faculty of Science, University of Tabuk, Tabuk, 47913 Saudi Arabia

**Keywords:** Glass, Melting quench technique, Gamma attenuation, Geant4 simulation, Materials science, Physics

## Abstract

The purpose of this work is to prepare glass samples with the chemical formula xPbO_2_-23BaO-10CaO-(65-x)B_2_O_3_-2Y_2_O_3_ (x = 10, 13, 16 and 19 mol%) using the usual melt quenching technique. These glasses are intended to be used as shielding materials designed to protect against ionizing radiation. Following the experimental measurement of the mass attenuation coefficient (G_MAC_), which was done to explore the photon shielding capabilities of the prepared samples. The findings were then compared with the values that were estimated from the Phys-x database and simulated using Geant4 code. The experimental findings that were obtained demonstrated a satisfactory connection with the data that was obtained from both Phy-x and Geant4. Based on the observed G_MAC_, other radiation shielding characteristics were computed for each of the glass that were investigated. These parameters included the linear attenuation coefficient (G_LAC_), the half-value layer (G_HVL_), and the mean free path (G_MFP_). Considering the data presented here, it seems that PBCBY-4 samples have the potential to be advantageous for use in shielding applications against ionizing radiation.

## Introduction

Radiation therapy is a treatment targeting the destruction of cancer cells. It involves the use of high-energy radiation beams to destroy cancer cells and tumors. The therapy aims to eliminate cancer cells, relieve symptoms, and prevent recurrence after treatment to improve the general quality of life of patients. Although Radiation is applicable in various ways. For example, it is used in medical facilities for sterilizing medical equipment, diagnosis, and treatment of various diseases. In industries for food preservation, and extension of shelf-life by reducing spoilage. In archaeology for age determination of artifacts and organic materials, and in nuclear plants for generating electricity^1,2^.

Its effect causes destruction to individuals, plants, and the environment. In individuals, negative effects can be mild or severe, leading to gene mutations, destruction of tissues, causing oxidative distress and DNA damage which directly inhibits growth by causing cell death. With severe effects leading to bone and marrow destruction, metastasis and development of cancer, Infertility through reproductive organ mutation and DNA alteration. Radiation sickness, nausea and vomiting, skin irritation, hair loss, fatigue, and dizziness are other common symptoms.

Materials like glass are used to absorb and reduce such effects^3–5^. Glass whose properties include high density, transparency which enables clear observation during procedures, durability, its ability to retain quality after recycling, great insulation properties, non-toxicity, chemical inertness, and its ability to incorporate metallic oxides qualifies it as an effective shielding material^6–10^. Metals such as lead, barium, bismuth, and others improve the overall shielding property of glass. Lead with high density and a high atomic number interacts with radiation rays and reduces their energy. Bismuth, commonly used as an alternative to lead possesses similar shielding properties to lead, making it highly effective for radiation attenuation. Barium in glass increases the mass attenuation coefficient and enhances its radiation shielding performance. This stems as a result of its high atomic number and density^11–14^.

Incorporating metallic oxides like lead oxide and barium oxide increases the shielding properties of glass; Lead oxide increased composition in glass directly increases a glass are shielding ability. Barium oxide increases the overall density of glass serving as an inhibitor for radiation. Boron trioxide, when incorporated with barium oxide increases the clarity and strength in glass, therefore, enhancing the radiation shielding proficiency^15^.

Further enhancement is obtained by introducing metallic oxides like yttrium. Yttrium oxide, a rare element with a melting point of 2,410 degrees Celsius is a highly corrosion resistant metal with low toxicity that exists in cubic, hexagonal, and monoclinic forms^16^. It enhances radiation attenuation by reducing the half value layer (HVL), and mean free path of glass while increasing the effective atomic number (Z_eff_), and electron density thereby increasing radiation attenuation, implying that glasses containing PbO_2_-BaO-B_2_O_3_-Y_2_O_3_ composition will have increased density, high atomic number, increased strength, durability, and effective shielding properties than those with other compositions.

Using experimental techniques for the assessment of a material’s radiation attenuation is crucial for real-life measurement of parameters like Half Value Layer (HVL), mean free path (MFP), effective atomic number (Z_eff_), linear attenuation coefficient, etc^17–20^. This study therefore experimentally investigated the radiation shielding ability of glass system composing of PbO_2_-BaO-CaO-B_2_O_3_-Y_2_O_3_.

## Materials and methods

**Fig. 1:**
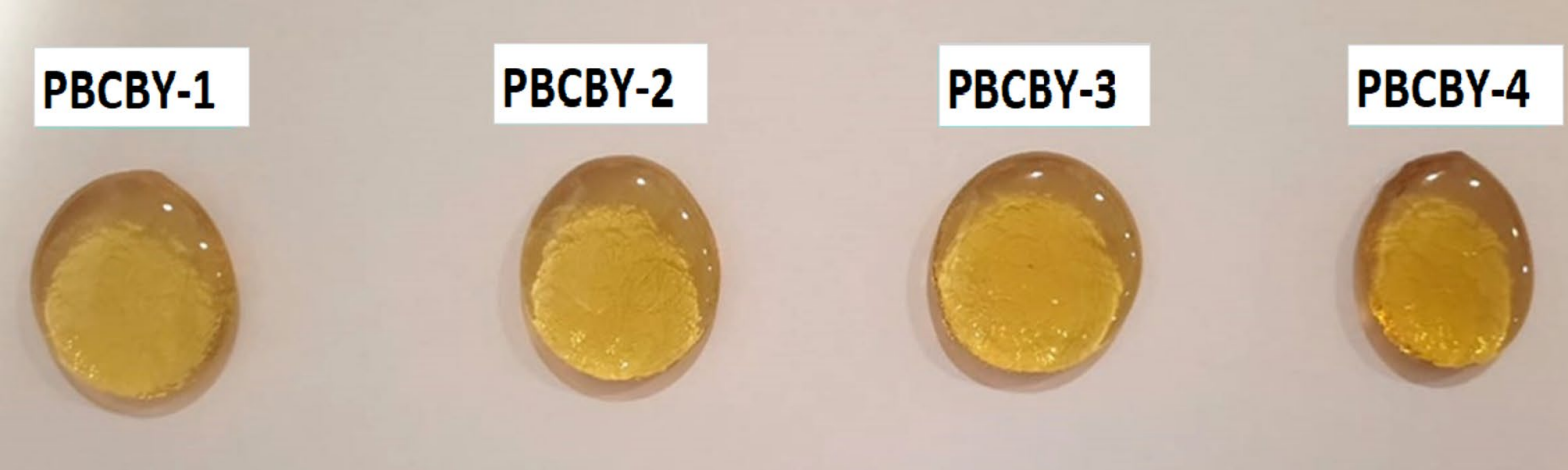
Image of fabricated glass samples.

The investigated glasses possess the general formula: xPbO_2_-23BaO-10CaO-(65-x)B_2_O_3_-2Y_2_O_3_, (x ranges from 0 to 9 mol% in 3 mol% increments). The melt quenching method was employed for their fabrication. In this investigation, the Y_2_O_3_ was sourced from Hebei Suoyi New Material Technology company, China, and the other oxides (with purity > 99.5% ) used in the preparation were obtained from Loba Cheme PVT. Ltd, India. After specific weighing, an electric furnace (Nabertherm model) was utilized to melt the chemicals in an alumina crucible for 40–50 min at 1100 °C. To establish glass samples that were properly homogeneous, the melts were thoroughly rotated several times. The sample melts were cast on a stainless-steel plate. The glasses were then moved into another furnace for 4 h at 350 °C (annealing process). Figure 1 shows the fabricated glass composites.

**Fig. 2:**
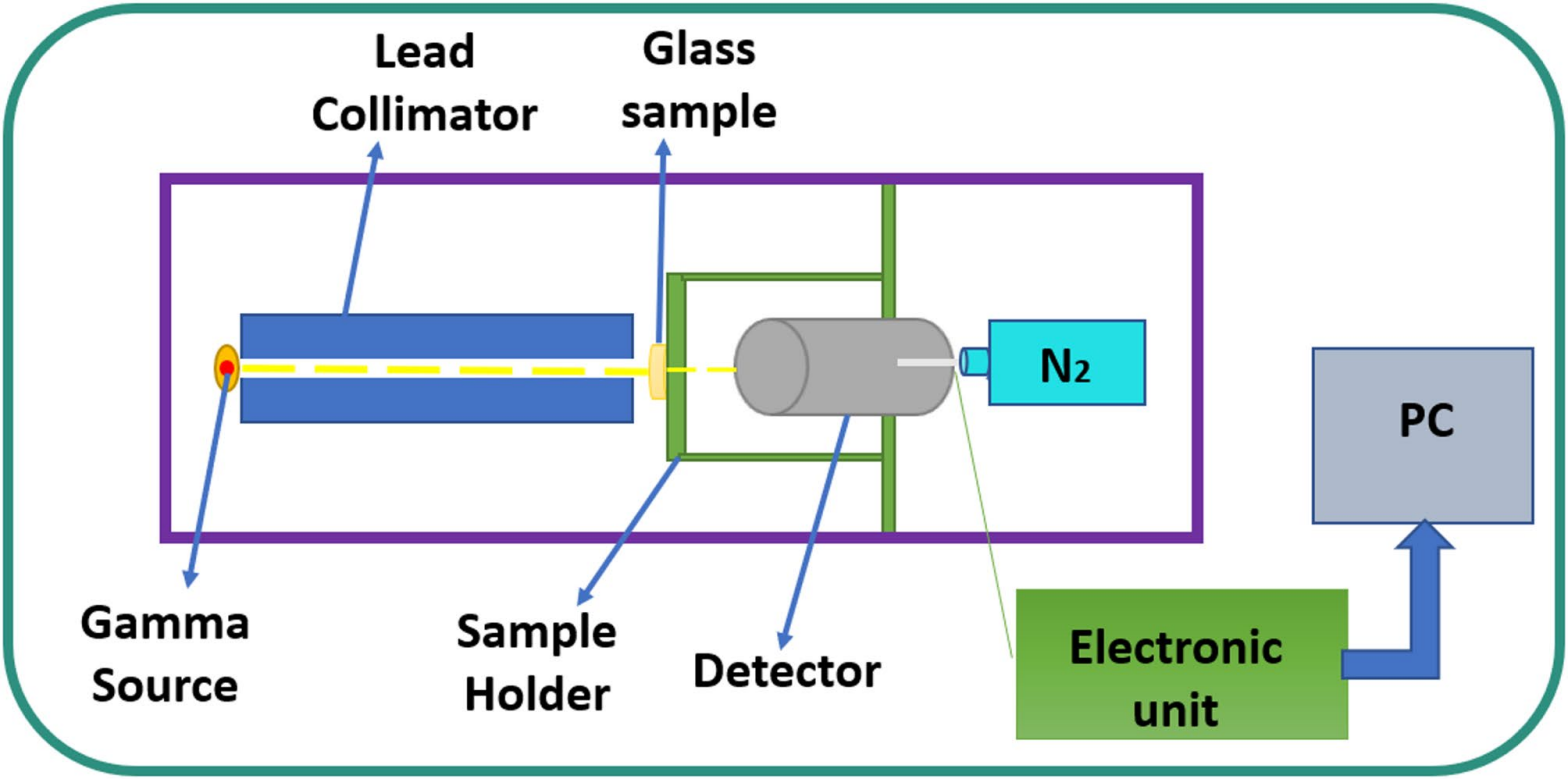
Experimental gamma-ray attenuation measurements.

In this work, experimental measurements were performed to determine the gamma-ray attenuation coefficients of the prepared PBCBY-glass samples. A high-pure germanium (HPGe) detector (Which form spectra analyzed using Genie2000, an integrated spectra collection and measurement software from Canberra Industries.) and standard radioactive sources (SRS) with different photon energies were used (The initial activity ~ 40 kBq on 1 September 1998 for three gamma sources used). The detector relative efficiency and energy resolution were 24% and 1.69 keV at energy line 1333 keV, respectively. The SRS were ^241^Am (emits gamma-line 60 keV), ^137^Cs (emits gamma-line 662 keV), ^60^Co (emits gamma-lines 1173 and 1333 keV), The HPGe detector was carefully calibrated to ensure the reliability of the results, and the position of the PBCBY-glass sample was calibrated between the SRS and the HPGe-detector and placed axially as shown in Fig. 2 using narrow beam technique, since the lead-collimator was used. A fixed geometry was used in all measurements, with a source–sample distance of 12 cm and a sample–detector distance of 4 cm to ensure reproducibility and minimize geometric uncertainties. The time period of measurement was not constant for all sources; rather, each spectrum was recorded using an optimized recording time to achieve the lowest possible statistical uncertainty in the net peak area (≤ 1%), depends on the energy and strength of gamma source. the average time for ^241^Am and ^137^Cs 1800 s, while for ^60^Co around 3600 s or more. The measurements were repeated multiple times and the average value was taken to reduce the error. The glass linear attenuation coefficient (G_LAC_) was evaluated by determining the photo-peak area in the absence of the PBCBY-glass which represents the intensity (I_o_), and determining the photo-peak area in the presence of the PBCBY-glass which represents the intensity (I) and the formula was reported in Table 1. The uncertainty in the measured LAC and other attenuating factors such as glass mass attenuation coefficient (G_MAC_), glass half value layer (G_HVL_) and glass mean free path (G_MFP_) were calculated according to equation in Table 1^21,22^.

**Table 1:** Radiation shielding parameters and related equations.

Radiation shielding parameters	Equations	Descriptions
Linear attenuation coefficient	$\:{G}_{LAC}=-\frac{Ln\left(\frac{I}{{I}_{^\circ\:}}\right)}{X}$	I_o_ is the gamma line intensity detected without the glass sample present during the measurements, I is the gamma line intensity detected with the glass sample present during the measurements, and X is the glass thickness.
Uncertainty in linear attenuation coefficient	$\:\varDelta\:\:LAC=\frac{1}{x}\sqrt{{\left(\frac{\varDelta\:I}{I}\right)}^{2}+{\left(\frac{{\varDelta\:I}_{0}}{{I}_{0}}\right)}^{2}+{(\mathrm{l}\mathrm{n}\frac{I}{{I}_{0}})}^{2}{\left(\frac{\varDelta\:x}{x}\right)}^{2}}$	$\:\varDelta\:I=\sqrt{I},\:\varDelta\:{I}_{o}=\sqrt{{I}_{0}}\:,\:$ *Δx* is the uncertainty in thickness
Mass attenuation coefficient	$\:{G}_{MAC}=\frac{{G}_{LAC}}{\rho\:}$	ρ is the glass density.
Half-value layer	$\:{G}_{HVL}=\frac{Ln\left(2\right)}{{G}_{LAC}}\:\left(cm\right)$	
Mean free path	$\:{G}_{MFP}=\frac{1}{{G}_{LAC}}\:\left(cm\right)$	
Transmission factor	$\:TF=\left({e}^{-{X\:G}_{LAC}}\right)\times\:100\:\left(\%\right)$	X is the glass thickness
Radiation protection efficiency	$\:RPE=(100-TF)$	

To compare the experimental results, a simulation model was built using Geant4 (GEometry ANd Tracking) version 10.3.p03, a Monte Carlo-based tool widely used for simulating particle-matter interactions. Geant4 accurately represents the geometric structure and physical properties of materials, helping to derive theoretical values for attenuation coefficients^23–26^. In addition, the Phy-X/PSD online software ((https://phy-x.net/module/physics/shielding/) was used to calculate radiation shielding coefficients based on oxide composition and density^27,28^. The results obtained from experimental measurements, simulations, and theoretical models were analyzed and compared to evaluate the radiation attenuation efficiency of the PBCBY-glasses.

## Results and discussion

**Fig. 3:**
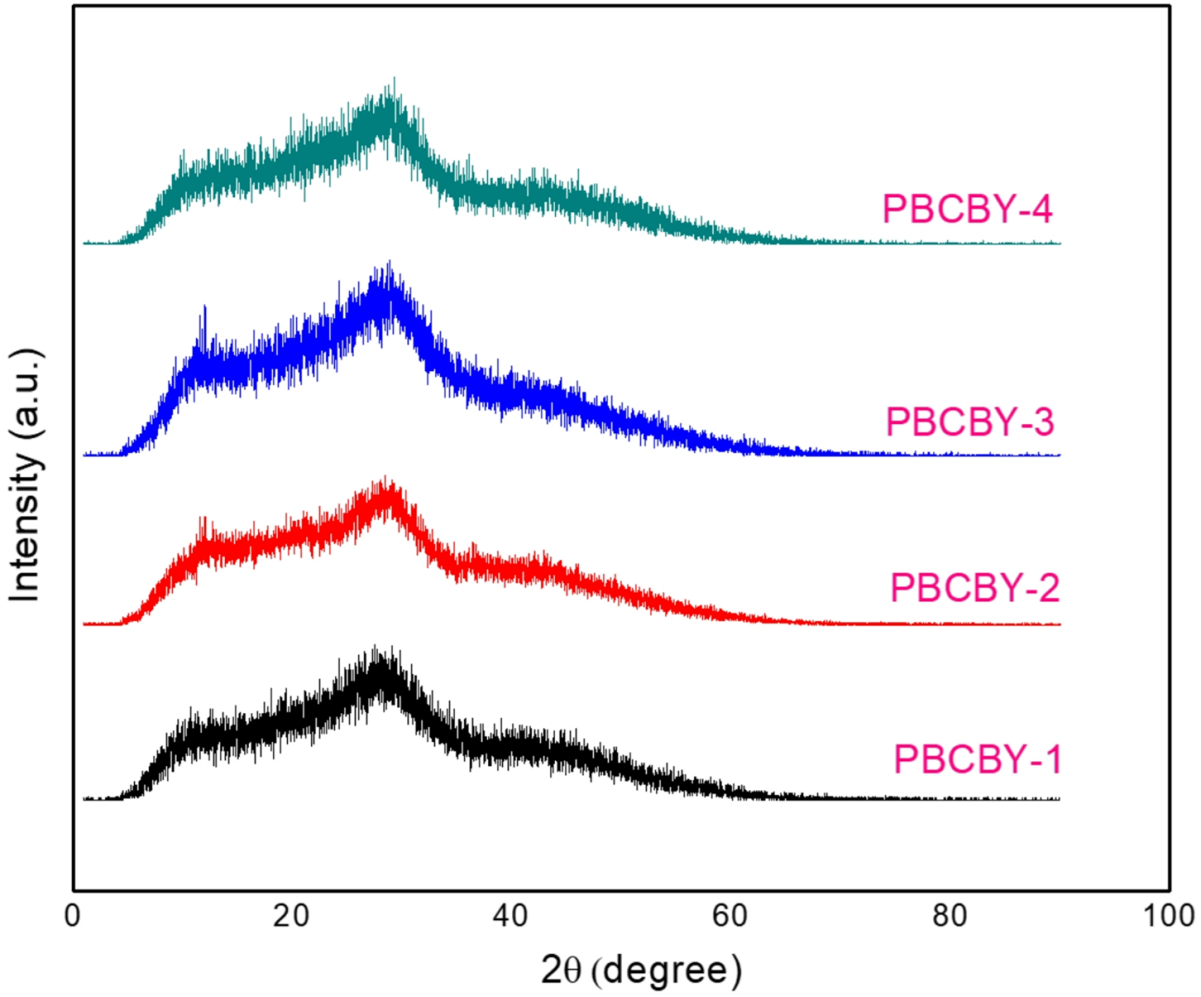
XRD analysis of PBCBY-glass composites.

For glass nature, the X-ray diffraction (XRD) patterns of PBCBY-glass composites prepared with different PbO_2_ contents is shown in Fig. 3. All samples exhibit a broad, diffuse halo centered approximately in the 2θ ≈ 20–35° range, with a complete absence of sharp Bragg diffraction peaks. This characteristic confirms the amorphous (glassy) nature of the studied samples. The similarity in the overall shape of the diffraction patterns indicates that increasing the PbO₂ content from PBCBY-1 to PBCBY-4 does not induce crystallization but rather slightly modifies the glass lattice structure. The preservation of the amorphous phase in all formulations confirms the successful fabrication of a homogeneous borate-based glass suitable for radiation shielding applications.

The mass attenuation coefficient (G_MAC_) for the examined samples 10PbO_2_-23BaO-10CaO-55B_2_O3-2Y_2_O_3_ (PBCBY-1), 13PbO_2_-23BaO-10CaO-52B_2_O3-2Y_2_O_3_ (PBCBY-2), 16PbO_2_-23BaO-10CaO-49B_2_O_3_-2Y_2_O_3_ (PBCBY-3), and 19PbO_2_-23BaO-10CaO-46B_2_O_3_-2Y_2_O_3_ (PBCBY-4) was determined experimentally using the gamma spectra from Am-241, Cs-137, and Co-60 sources. The equations and nomenclature that are associated with the shielding parameters are shown in Table 1^29^. The findings are then compared with the computed and simulated G_MAC_ values using Phy-X software and Geant4 code (Table 2). The percentage differences between the measured and estimated G_MAC_ (Δ_1_) and the measured and simulated G_MAC_ (Δ_2_) were then computed and shown in Table 2. Because of this, we have seen that the values measured in the experiment are in excellent agreement with the values that were calculated and simulated across the board for all energies. When it comes to numerical data, the experimental G_MAC_ for the PBCBY-1 sample at 0.662 MeV is measured to be 0.081cm^2^/g. This value is found to be in excellent agreement with the estimated value of 0.083 cm^2^/g (Δ_1_ = 2.79%) and simulated value 0.083 (Δ_2_ = 2.36%) cm^2^/g that was acquired by Phy-x and Geant4, respectively. A high degree of correlation exists between the (G_MAC_)_Exp_ values and both the (G_MAC_)_Phy−x_ and (G_MAC_)_Geant4_ values, as shown by the percentage difference between the (G_MAC_)_Exp_ values and both the (G_MAC_)_Phy−x_ and (G_MAC_)_Geant4_ values. Like the previous example, when we replace 19% of PbO_2_ on the B_2_O_3_ site at 662 keV, for instance, the G_MAC_ values that were obtained by experimental, Phy-x, and Geant4 were found to be 0.087, 0.087, and 0.087 cm^2^/g, respectively, with a percentage difference of around 0.40 and 0.49%. The validation of the experimental setup and both calculation and simulation of other ionising radiation shielding parameters may be accomplished with the help of the findings that are shown in Table 2. These results give proof that the G_MAC_ values of the samples that were tested are correct.

**Table 2:** Experimental, theoretical, and simulated mass Attenuation coefficient results for samples.

Sample code	Energy (keV)	Mass attenuation coefficient (G_MAC_, cm^2^/g)			Δ_1_	Δ_2_
		Phy-x	Geant4	Exp		
PBCBY-1	59.54	3.760	3.754	3.527 ± 0.032	6.19	6.43
	662	0.083	0.083	0.081 ± 0.009	2.79	2.36
	1173	0.057	0.056	0.057 ± 0.006	0.43	1.68
	1333	0.053	0.054	0.052 ± 0.006	1.84	4.59
PBCBY-2	59.54	3.871	3.838	3.675 ± 0.038	5.06	4.45
	662	0.085	0.083	0.081 ± 0.009	3.99	2.27
	1173	0.057	0.056	0.055 ± 0.006	3.01	0.83
	1333	0.053	0.053	0.052 ± 0.006	1.10	0.44
PBCBY-3	59.54	3.972	3.829	3.740 ± 0.041	5.86	2.40
	662	0.086	0.084	0.082 ± 0.009	4.67	2.44
	1173	0.057	0.056	0.055 ± 0.006	3.60	1.20
	1333	0.053	0.052	0.052 ± 0.006	3.07	0.43
PBCBY-4	59.54	4.065	4.018	3.846 ± 0.043	5.40	4.49
	662	0.087	0.087	0.087 ± 0.010	0.40	0.49
	1173	0.058	0.057	0.058 ± 0.006	0.65	1.82
	1333	0.053	0.052	0.052 ± 0.006	2.77	1.22

$$\:{\varDelta\:}_{1}=\frac{{\left({G}_{MAC}\right)}_{Phy-x}-{\left({G}_{MAC}\right)}_{Exp}}{{\left({G}_{MAC}\right)}_{Phy-x}}\times\:100$$

$$\:{\varDelta\:}_{2}=\frac{{\left({G}_{MAC}\right)}_{Geant4}-{\left({G}_{MAC}\right)}_{Exp}}{{\left({G}_{MAC}\right)}_{Geant4}}\times\:100$$

**Fig. 4:**
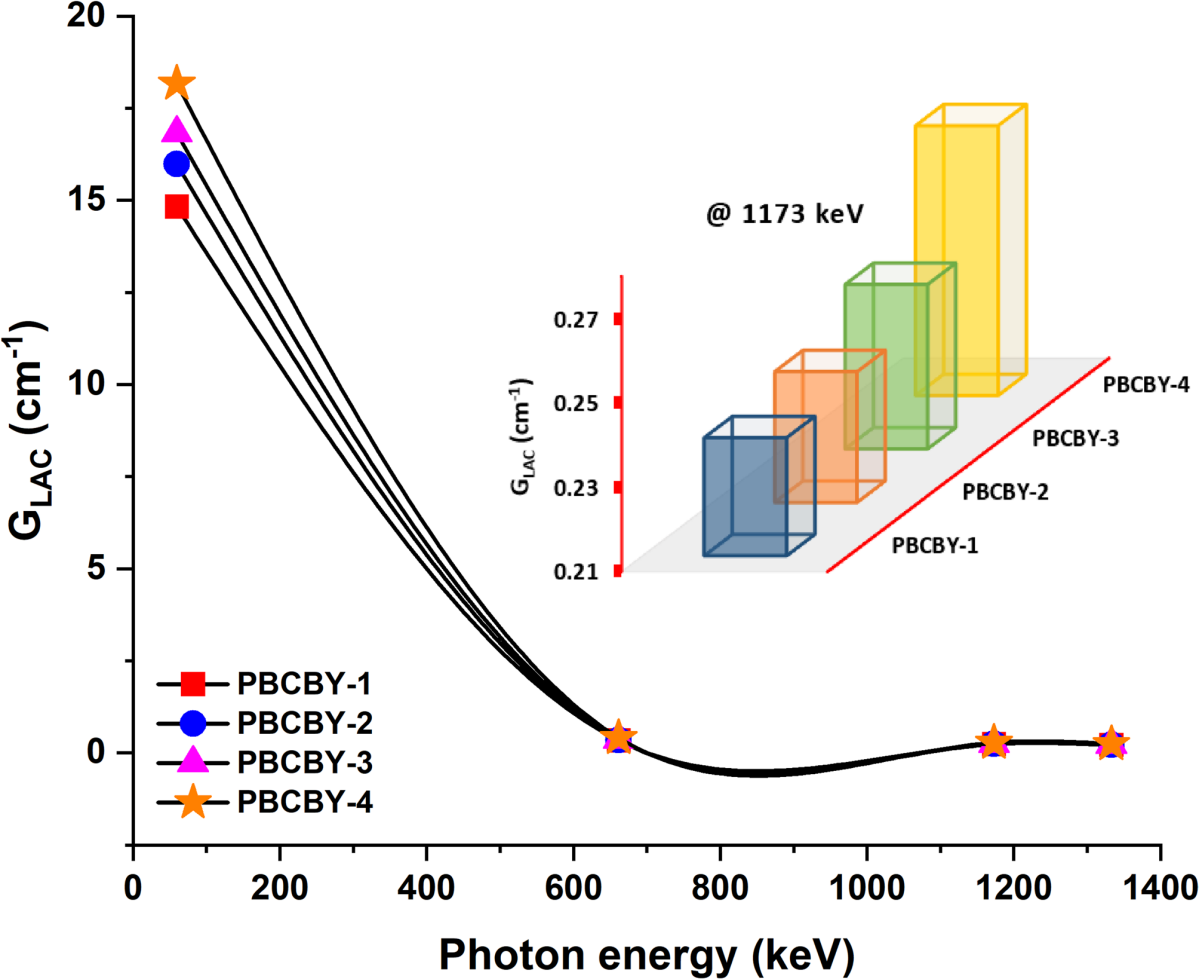
Linear attenuation coefficient (G_LAC_) as a function on photons energy for all prepared glass samples.

The linear attenuation coefficient (G_LAC_) is a measure that quantifies the amount of attenuation that a photon beam experiences as it passes through a material. This attenuation is highly impacted by factors such as the density of the material and the energy of the photons. The change of G_LAC_ as a function of incoming photon energy is shown in Fig. 4 for the PBCBY-1, PBCBY-2, PBCBY-3, and PBCBY-4 samples that were manufactured and studied. A decrease in G_LAC_ values that is exponential in nature was determined by us as the energy of the incoming photons increased. To put it another way, the value of G_LAC_ would fall as the amount of energy would increase. For example, the G_LAC_ of the sample containing 19% PbO_2_ decreased from 18.171 cm^− 1^ at 59.54 keV to 0.411, 0.274, and 0.245 cm^− 1^ at 662, 1173, and 1333 keV, respectively. Furthermore, we observed that the G_LAC_ values improved when the addition of PbO_2_ was added. Compared to the higher energies, this improvement is far more substantial. For instance, when the energy was 59.54 keV, 14.829, 15.982, 16.832, and 18.171 cm-1 are the G_LAC_ values of PBCBY-1 (10% of PbO_2_), PBCBY-2 (13% of PbO_2_), PBCBY-3 (16% of PbO_2_), and PBCBY-4 (19% of PbO_2_) samples, respectively. When the energies are increased, the G_LAC_ values that are found in each sample become more like one another. Among all the samples that were prepared, the PBCBY-4 sample that had the greatest concentration of PbO_2_ (x = 19%) had the best G_LAC_ values.

It is evident that the G_LAC_ diminishes as the incident gamma energy increases. The increase in G_LAC_ values at lower energy is attributable to the photoelectric effect^30–32^. At low energies, the attenuation coefficients are significantly dependent on the energy of the input photon (E^− 3.5^), as elucidated by the photoelectric absorption relationship. Consequently, the lead samples, owing to their elevated atomic number, exhibited enhanced photoelectric absorption at low incoming gamma energies. The existence of a high atomic number may have led to a rise in the density of the samples that were created, which might be linked to the increase in the likelihood of interactions between photons and atoms. As a result of this, the strength of the incoming gamma radiation is reduced, which leads to the attenuation of the samples. In addition, the energy transfer is significantly dependent on Compton scattering for energies of a mid-level range. Compton scattering, in turn, is dependent on the energy (E^− 1^) of the photon that is incident, with the energy of the photon being inversely proportional to the energy. In addition to this, the phenomena of Compton scattering, which is a kind of inelastic scattering, reduces the intensity of the gamma radiation that is incident by transferring the energy that is required for the recoiling process. Furthermore, it is important to point out that the material that has a high atomic number is beneficial in the attenuation of gamma radiation that is at an intermediate energy level. In addition, the G_LAC_ being reduced at the higher energy level may be attributed to the fact that energy E is dependent on the atomic number, which is in turn dependent on Z^2^, and this leads to the occurrence of pair production. Therefore, the existence of particles for larger interactions of energy might certainly result in the attenuation of incoming gamma radiation, as it could be understood^33,34^.

**Fig. 5:**
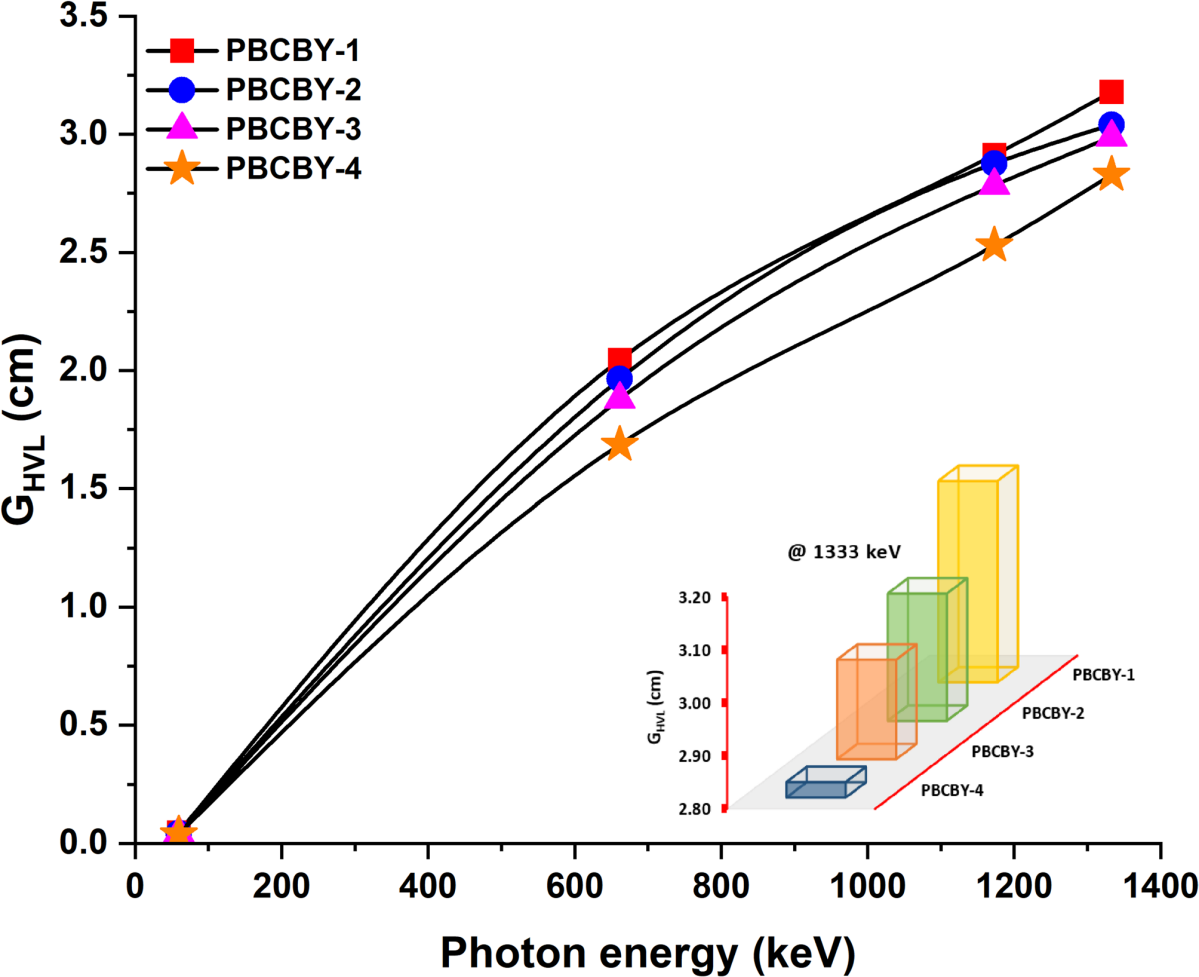
Half value layer (G_HVL_) as a function on photons energy for all prepared glass samples.

The half-value layer (G_HVL_) is a term that is used to define the required thickness of a material or composition to reduce the intensity of the ionising radiation to half of its original value. The variance of the G_HVL_ for the prepared PBCBY-1, PBCBY-2, PBCBY-3, and PBCBY-4 samples is shown in Fig. 5, including how it varies with the energy. We found that the G_HVL_ values increased as the amount of energy increased. For example, 0.04, 1.69, 2.53, and 2.83 cm are G_HVL_ values of PBCBY-4 (19% of PbO_2_) sample at 59.54, 662, 1173, and 1333 keV, respectively. It has been noticed that the G_HVL_ values at 59.54 keV are quite low. This might be ascribed to the comparatively high G_MAC_ values at these values (see Table 2), which provides evidence that a PBCBY-1, PBCBY-2, PBCBY-3, and PBCBY-4 samples that is relatively thin can be used to absorb low-energy photons. On the other hand, the G_HVL_ decreases when the concentration of PbO_2_ increases. For example, at 662 keV, 2.04, 1.96, 1.88, and1.69 cm are G_HVL_ values of PBCBY-1 (10% of PbO_2_), PBCBY-2 (13% of PbO_2_), PBCBY-3 (16% of PbO_2_), and PBCBY-4 (19% of PbO_2_) samples, respectively. Furthermore, these findings provide additional evidence that the substitution of PbO_2_ on the B_2_O_3_ side in PBCBY-1, PBCBY-2, PBCBY-3, and PBCBY-4 samples is of critical relevance.

**Fig. 6:**
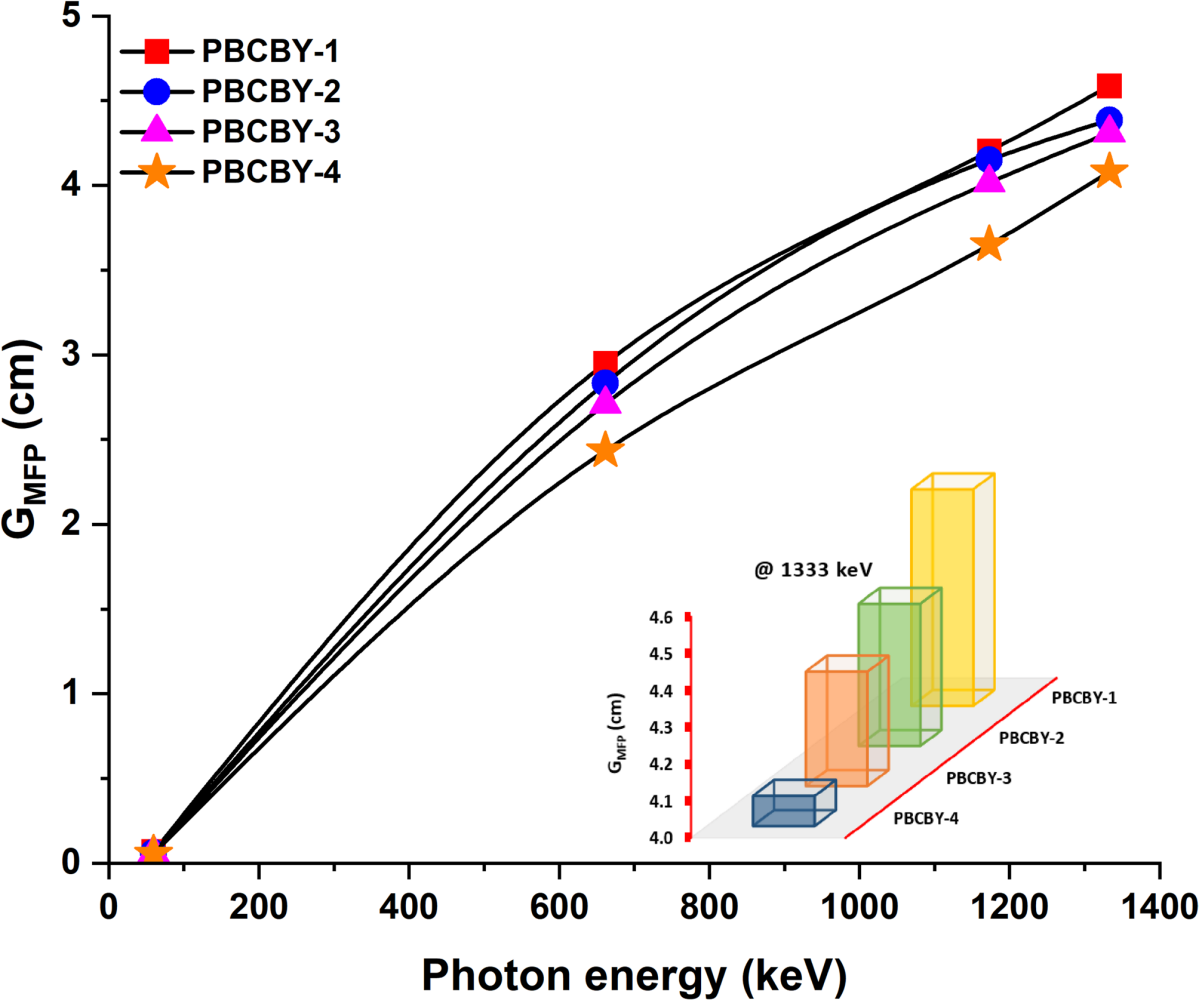
Mean free path (G_MFP_) as a function on photons energy for all prepared glass samples.

**Fig. 7:**
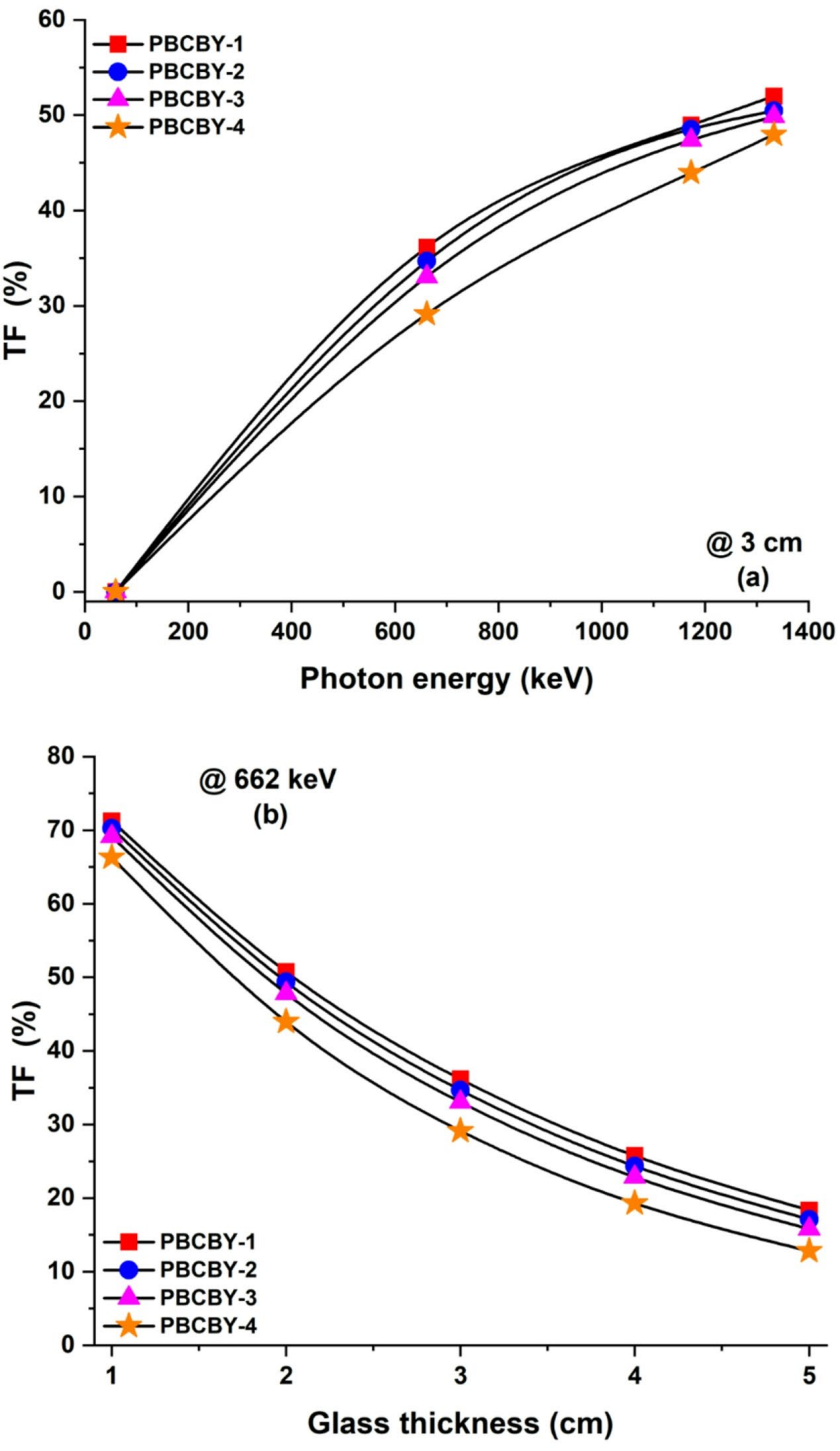
(**a**) The transmission factor (TF, %) for 3 cm of prepared samples at different energies. (**b**) The TF at 0.662 keV incident photons on prepared samples versus sample thickness.

A photon in a shielding composition will travel a certain distance between two subsequent collisions. This distance is defined by the mean-free path (G_MFP_), which is an essential factor in ionising radiation shielding. A shielding material has an G_MFP_ that is equal to the inverse of the G_LAC_. On display in Fig. 6 are the values of the G_MFP_ as a function of the energy that is incident. Because the values of the estimated G_MFP_ rise with rising photons energy, the behaviour of the G_MFP_ is identical to that of the G_HVL_. For example, 0.06, 2.43, 3.65, and 4.08 cm are G_MFP_ values of PBCBY-4 (19% of PbO_2_) sample at 59.54, 662, 1173, and 1333 keV, respectively, this is a significant increase. Additionally, as can be observed from Fig. 7, an increase in the PbO_2_ content results in a decrease in the G_MFP_ values. For example, at 662 keV, 2.95, 2.83, 2.71, and 2.43 cm are G_MFP_ values of PBCBY-1 (10% of PbO_2_), PBCBY-2 (13% of PbO_2_), PBCBY-3 (16% of PbO_2_), and PBCBY-4 (19% of PbO_2_) samples, respectively.

The transmission factor (TF) was computed so that we could evaluate the radiative attenuation qualities of the samples that had been successfully prepared. The number of gamma rays that can pass through the glass that is being utilized as a shield is indicated by this characteristic. A TF value that is lower indicates that shielding capabilities are improved. Figure 7**(a)** illustrates the TF for the 3 cm of the prepared samples that have been processed at a variety of energies. Our conclusion was that the shielding material would allow more radiation to flow through it if the energy of the radiation was increased. For example, The TF for PBCBY-4 glass sample increases from 2 × 10^22^ to 48% when the photon energy increases from 59.54 to 1333 keV. PBCBY-1, PBCBY-2, PBCBY-3, and PBCBY-4 samples exhibit behaviour that is comparable to one another. Additionally, it is obvious that increasing the quantity of PbO_2_ in the sample results in a reduction in the number of photons that can pass through the shielding material provided that the sample thickness remains the same. It may be said that samples that have a greater amount of PbO_2_ are more suitable for usage as a shielding material. Figure 7**(b)** illustrates the TF for photons at 662 keV that are incident at variations in the thickness of the samples. Based on the observations, it can be concluded that the TF value falls as the sample thickness increases. For example, 71, 50, 36, 25, and 18% are TF values of PBCBY-1 sample at 1, 2, 3, 4, and 5 cm, respectively. PBCBY-1, PBCBY-2, PBCBY-3, and PBCBY-4 samples exhibit behaviour that is comparable to one another. A further conclusion that can be drawn is that the TF decreased as the amount of PbO_2_ in the PBCBY-1, PBCBY-2, PBCBY-3, and PBCBY-4 samples increased. This indicates that the penetration rate decreases as the amount of PbO_2_ substitution increases.

**Fig. 8:**
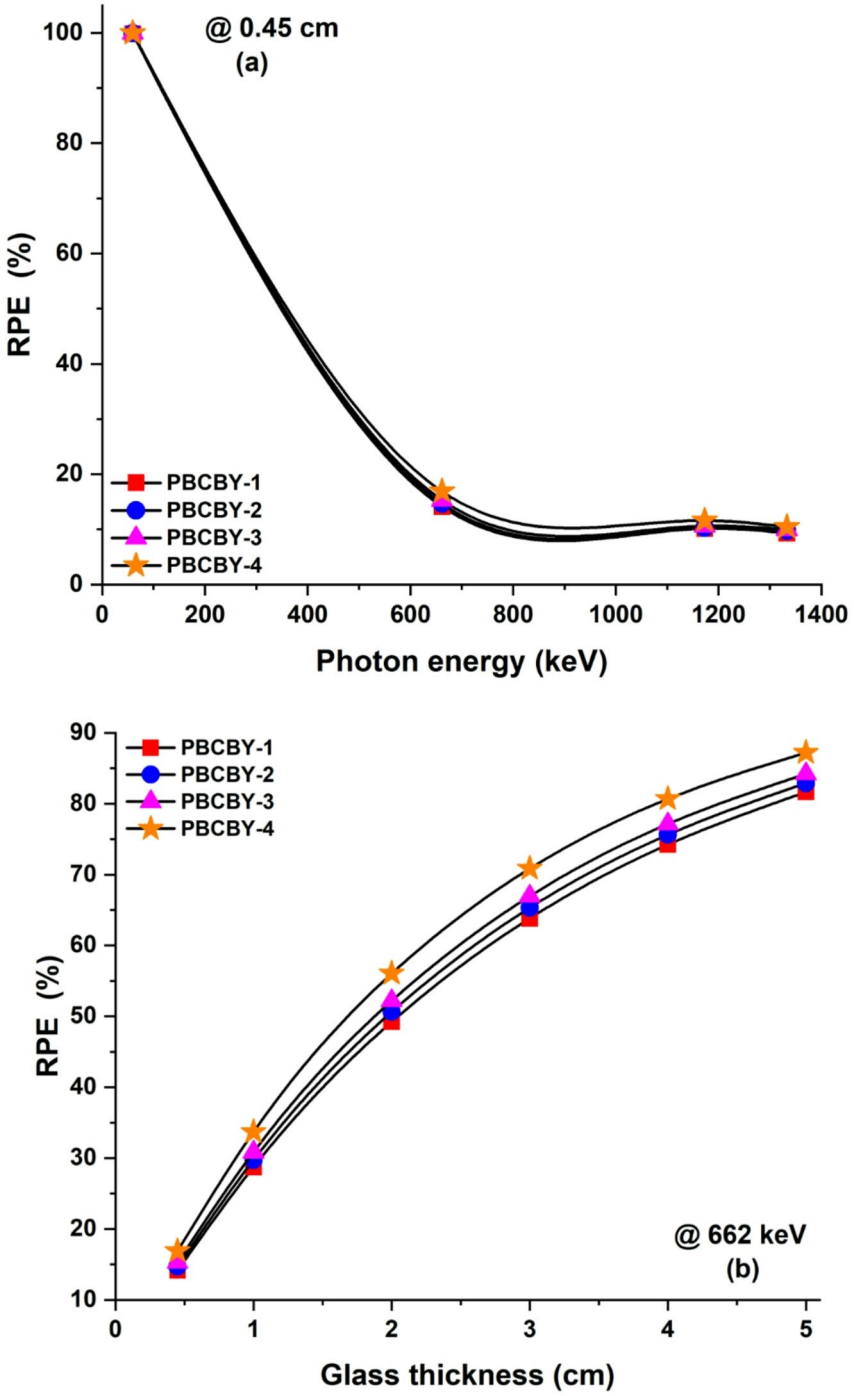
(**a**) The radiation protection efficiency (RPE, %) for 3 cm of prepared samples at different energies. (**b**) The RPE at 0.662 keV incident photons on prepared samples versus sample thickness.

To determining how effectively the processed samples function as photon beam shielding materials, we also computed the radiation protection efficiency (RPE). RPE of PBCBY-1, PBCBY-2, PBCBY-3, and PBCBY-4 samples at normalized thickness (0.45 cm) is shown in Fig. 8**(a)**, which makes use of the specific energy range. It came to our attention that the RPE is inversely proportional to the energy of the photon that is incident onto it. A representation of the RPE of the prepared glasses that were investigated is shown in Fig. 8**(b)** when they were subjected to 662 keV photons at different sample thicknesses. At normalized thickness (0.45 cm) of PBCBY-1, PBCBY-2, PBCBY-3, and PBCBY-4 samples are subjected to 662 keV photons, the attenuation of the samples is 14.15, 14.69, 15.30, and 16.89%, respectively. While, at 5 cm of PBCBY-1, PBCBY-2, PBCBY-3, and PBCBY-4 samples are subjected to 662 keV photons, the attenuation of the samples is 81, 82, 84, and 87%, respectively. The results of this investigation provide credence to the efficiency of the system that was studied for photon attenuation. Finally, the fact that PBCBY-4 glass had the lowest set of G_HVL_ values found suggested that it has better attenuation qualities in comparison to other compositions. Table 3 shows the comparison of G_HVL_ of prepared glass (PBCBY-4) with other shielding materials such as: glasses (S1, S2, S3, PCNKBi7.5, Pb20, PbG, S5^35–39^, concretes (OC, HSC, ILC, BMC, SSC^40^, and polymers (PbCl2(20%), 20% BaZrO3, NPW20, and Nb(15%)^41–44^. As listed in this table, G_HVL_ values of PBCBY-4 glass at 662, 1173, and 1333 keV are lower than the other shielding materials.

**Table 3:** Comparison of half value layer of prepared glass (PBCBY-4) with other shielding materials.

	662 keV	1173 keV	1333 keV
**PBCBY-4** **(This work)**	**1.69**	**2.53**	**2.83**
S1	4.48	5.87	6.27
S2	3.80	4.99	5.32
S3	3.06	4.04	4.31
PCNKBi7.5	3.92	5.14	5.49
Pb20	3.85	5.13	5.48
PbG	3.77	4.95	5.28
S5	3.36	4.51	4.82
OC	3.8	4.98	5.31
HSC	3.62	4.75	5.07
ILC	3.19	4.19	4.47
BMC	2.98	3.91	4.17
SSC	2.32	3.05	3.25
PbCl2(20%)	6.32	8.87	9.54
20% BaZrO3	6.20	8.24	8.24
NPW20	6.11	8.61	8.83
Nb(15%)	6.55	8.53	8.97

## Conclusion

The melting quench technique was utilized to synthesize a novel glass system, xPbO_2_-23BaO-10CaO-(65-x)B_2_O_3_-2Y_2_O_3_ (x = 10, 13, 16, 19 mol%). An experimental determination was made to measure the G_MAC_ of samples. This was followed by a comparison with the results acquired from the Phy-x program and Geant4 code at 59.54, 662, 1173, and 1333 keV. It has been determined that the experimental G_MAC_ for the PBCBY-4 sample (i.e., x = 19 mol%) at 662 keV is 0.087 cm^2^/g. This value is found to be in excellent agreement with the estimated value of 0.0873 and 0.0866 cm^2^/g that was acquired using Phy-x (0.4%) and Geant4 (0.49%), respectively. It was possible to get a good correlation between both theoretical and simulation and experimental G_MAC_ values. After that, several radiations shielding parameters, including LAC, HVL, MFP, TF, and RPE, were computed. In general, the capacity of the material to attenuate photons had a negative correlation with the amount of photon energy that was present. While this was going on, the shielding characteristics of the PBCBY-1, PBCBY-2, PBCBY-3, and PBCBY-4 samples were enhanced when PbO_2_ was added to them. These results provide evidence that the system under investigation is effective for the attenuation of photons and point to the possibility of using the PBCBY-4 sample for the purpose of photon shielding.

## Data Availability

The data presented in this study are available on request from the corresponding author.
